# Knockdown of Cytosolic Glutaredoxin 1 Leads to Loss of Mitochondrial Membrane Potential: Implication in Neurodegenerative Diseases

**DOI:** 10.1371/journal.pone.0002459

**Published:** 2008-06-18

**Authors:** Uzma Saeed, Lalitha Durgadoss, R. Khader Valli, Dinesh C. Joshi, Preeti G. Joshi, Vijayalakshmi Ravindranath

**Affiliations:** 1 Division of Molecular and Cellular Neuroscience, National Brain Research Centre, Nainwal Mode, Manesar, India; 2 Department of Biophysics, National Institute of Mental Health and Neurosciences, Bangalore, India; Chiba University Center for Forensic Mental Health, Japan

## Abstract

Mitochondrial dysfunction including that caused by oxidative stress has been implicated in the pathogenesis of neurodegenerative diseases. Glutaredoxin 1 (Grx1), a cytosolic thiol disulfide oxido-reductase, reduces glutathionylated proteins to protein thiols and helps maintain redox status of proteins during oxidative stress. Grx1 downregulation aggravates mitochondrial dysfunction in animal models of neurodegenerative diseases, such as Parkinson's and motor neuron disease. We examined the mechanism underlying the regulation of mitochondrial function by Grx1. Downregulation of Grx1 by shRNA results in loss of mitochondrial membrane potential (MMP), which is prevented by the thiol antioxidant, α-lipoic acid, or by cyclosporine A, an inhibitor of mitochondrial permeability transition. The thiol groups of voltage dependent anion channel (VDAC), an outer membrane protein in mitochondria but not adenosine nucleotide translocase (ANT), an inner membrane protein, are oxidized when Grx1 is downregulated. We then examined the effect of β-N-oxalyl amino-L-alanine (L-BOAA), an excitatory amino acid implicated in neurolathyrism (a type of motor neuron disease), that causes mitochondrial dysfunction. Exposure of cells to L-BOAA resulted in loss of MMP, which was prevented by overexpression of Grx1. Grx1 expression is regulated by estrogen in the CNS and treatment of SH-SY5Y cells with estrogen upregulated Grx1 and protected from L-BOAA mediated MMP loss. Our studies demonstrate that Grx1, a cytosolic oxido-reductase, helps maintain mitochondrial integrity and prevents MMP loss caused by oxidative insult. Further, downregulation of Grx1 leads to mitochondrial dysfunction through oxidative modification of the outer membrane protein, VDAC, providing support for the critical role of Grx1 in maintenance of MMP.

## Introduction

Mitochondria play a pivotal role in cell function both in terms of being the power centers of the cell as well as mediators of cell death through apoptosis. Mitochondrial dysfunction has been implicated in a variety of neurodegenerative disorders. For example, abnormalities in mitochondrial complex I have been observed in several infantile and childhood neurological disorders and in neurodegenerative diseases such as Parkinson's disease [1], [2] and motor neuron disease [3] while complex II dysfunction is seen in Huntington's disease [4], [5]. The mechanisms underlying the dysfunction and their role in neurodegeneration are not entirely clear although it is generally believed that oxidative stress is a key player in some of these events [6]. While a close association and synergistic interplay exists between oxidative stress, mitochondrial dysfunction and neurodegeneration, clear identification of the events being either causative or consequential is yet to emerge. Earlier studies with animal models of Parkinson's disease have shown that glutathione (GSH) loss and oxidative stress may precede complex I dysfunction [7] and further, the loss of complex I activity can be restored by thiol antioxidants [8]. These observations clearly point to the role of oxidative stress as a causative factor in complex I dysfunction.

β-N-oxalyl amino-L-alanine (L-BOAA, also known as β-N-oxalyl-α,β-diamino propionic acid, β-ODAP; [9]) is an excitatory amino acid that acts as an agonist for the AMPA sub-class of glutamate receptors [10], [11]. Ingestion of the chickling pea that contains L-BOAA as the sole source of cereal leads to the development of a type of motor neuron disease known as neurolathyrism. The pathological hallmark of this disease includes degeneration of anterior horn cells and loss of axons in the pyramidal tracts in lumbar spinal cord in humans. Oxidative stress and mitochondrial dysfunction are major contributors to L-BOAA induced toxicity [12], [13]. L-BOAA causes GSH loss and increase in protein-glutathione mixed disulfides (PrSSG) in lumbosacral cord of male mice [3] resulting in selective inhibition of mitochondrial complex I, a major component of the mitochondrial electron transport chain, due to oxidation of critical thiol groups [13].

Thiol disulfide oxido-reductases are a group of enzymes that catalyze disulfide interchange reactions including conversion of glutathionylated proteins (PrSSG) to protein thiols (PrSH). This class of enzymes include glutaredoxin [14], [15], thioredoxin and protein disulfide isomerase [16]. These enzymes involve two hydrogen donor systems, a thioredoxin system and a GSH dependent glutaredoxin system [17]. Glutaredoxin 1 (also known as thioltransferase; Grx1), a cytosolic thiol disulfide oxido-reductase isolated from calf thymus [18] and human placenta [19] shows a GSH-disulfide transhydrogenase activity [20] and specifically and efficiently reduces glutathionylated proteins to protein thiols [21], [22], whereas thioredoxin and protein disulfide isomerase have broad substrate specificities [23]. Glutaredoxin 1 senses cellular redox potential and catalyzes glutathionylation, an important redox regulatory mechanism in response to oxidative stress [24]. Grx1 is essential for maintenance of complex I function in normal conditions [25] and its upregulation is critical for recovery of complex I function following L-BOAA administration [13].

Grx1 is a cytosolic protein and therefore its ability to influence mitochondrial function in this manner is not anticipated. In the present study we have examined the role of Grx1 in maintenance of mitochondrial membrane potential by downregulating Grx1 expression using shRNA to Grx1 in cultured neuroblastoma cells. Further, we have also exposed cells to the excitatory amino acid L-BOAA and studied the interplay between Grx1 and mitochondrial membrane potential. We have earlier observed that constitutive expression of Grx1 is significantly higher in female brain regions as compared to the male brain. Downregulation of Grx1 in female mouse brain renders them vulnerable to L-BOAA mediated mitochondrial dysfunction in a manner similar to that seen in male mice [26]. We therefore studied the effect of estrogen on expression of Grx1 and the ability of estrogen to protect against mitochondrial dysfunction mediated by L-BOAA.

## Results

### Downregulation of Grx1 generates reactive oxygen species

**Figure 1:**
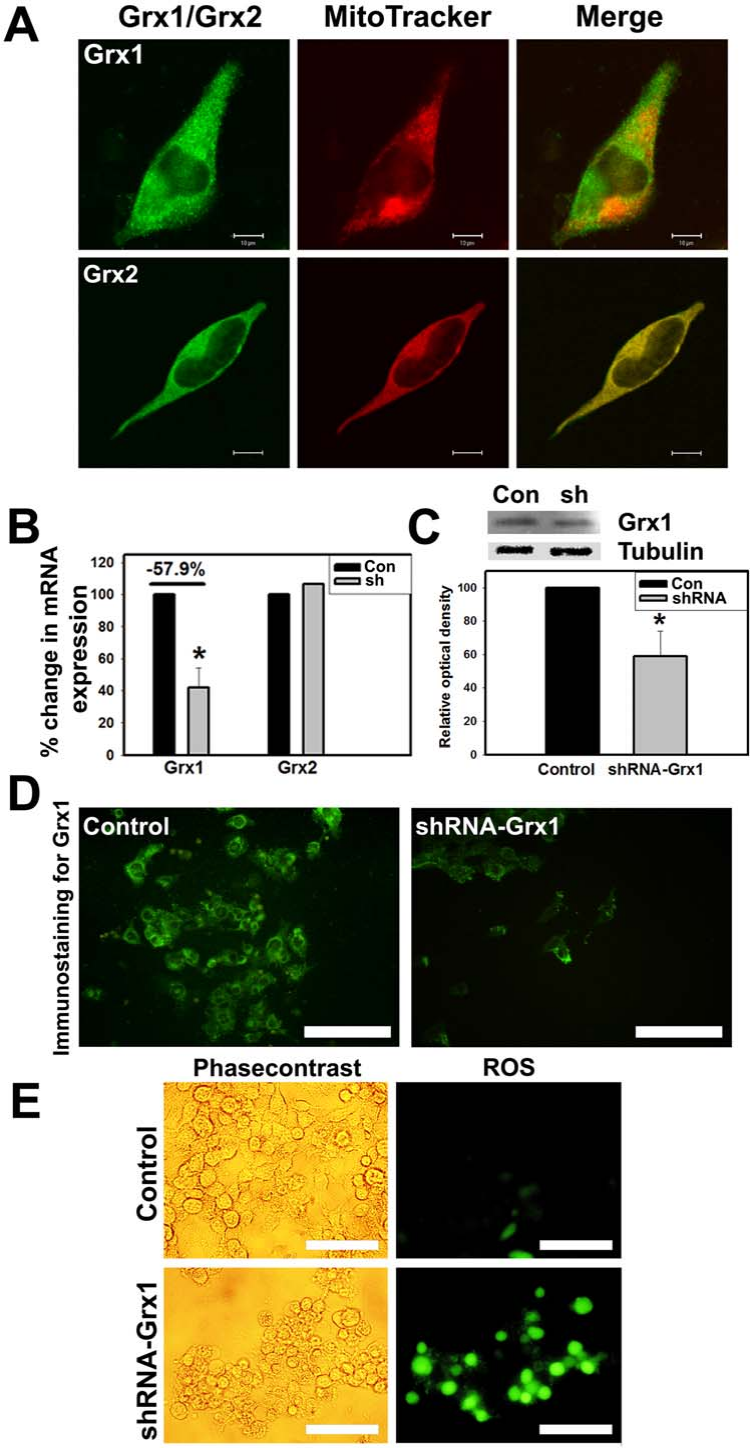
Localization of Grx1 in cytosol and generation of ROS following its knockdown in Neuro-2a cells. Cells were loaded with MitoTracker Deep Red 633 (500 nM) for 45 min before fixation and subsequent immunostaining for Grx1 or Grx2. Images were captured using LSM510 META in confocal microscope. (A) Grx1 (green) does not colocalize with MitoTracker Deep Red 633 (red) in Neuro-2a cells indicating its cytosolic localization, whereas mitochondrial glutaredoxin 2 (green; Grx2) colocalizes with MitoTracker Deep Red. Bar represents 10 µm. (B) Quantitation of knockdown of Grx1 as assessed by qRT-PCR, no change observed in Grx2 mRNA levels. Data is represented as mean±SEM from 3 independent experiments. Asterisks indicate values significantly different from controls (p<0.01). (C) Immunoblots depicting Grx1 protein levels in control (con) and knockdown using shRNA (sh) in Neuro-2a cells and densitometric quantitation of immunoblot after normalization with β-tubulin. Data is represented as mean±SEM from 3 independent experiments. Asterisks indicate values significantly different from controls (p<0.01). (D) Immunostaining for Grx1 in Neuro-2a cells transfected with empty vector or shRNA to Grx1. Bar represents 100 µM. (E) Downregulation of Grx1 using shRNA in Neuro-2a cells enhances ROS production as seen by increased H_2_DCFDA staining. Bar represents 100 µm.

Grx1 is a cytosolic protein disulfide oxido-reductase, while Grx2, another member of dithiol glutaredoxin enzyme family is localized essentially in the mitochondria and the nucleus [27]–[30]. In order to confirm the localization of Grx1 in the cytoplasm, Neuro-2a cells were loaded with MitoTracker (Deep Red 633) and immunostained with antibody to Grx1 or Grx2. We observed that Grx1 did not colocalize with MitoTracker confirming its cytoplasmic localization, unlike Grx2, which completely colocalized with MitoTracker (Fig. 1A). In order to generate cells in which Grx1 was downregulated, we transiently transfected Neuro-2a cells with shRNA to Grx1 and observed a consistent knockdown of Grx1 after 72 hr. The immunoblot of Grx1 from cells in which Grx1 was knocked down is shown (Fig. 1C). We used qRT-PCR to quantify the altered expression of Grx1 and Grx2 in these cells (Fig. 1B). While expression of Grx1 was significantly reduced in cells transfected with shRNA, Grx2 levels were unaffected indicating the specificity of the shRNA. The alteration in the expression of Grx1 was also validated by immunohistochemistry (Fig. 1D). Downregulation of Grx1 leads to generation of reactive oxygen species as seen by H_2_DCFDA (2′,7′-dichlorodihydrofluorescein diacetate) staining (Fig. 1E).

### Optimum downregulation of Grx1 leads to alteration in mitochondrial membrane potential

**Figure 2:**
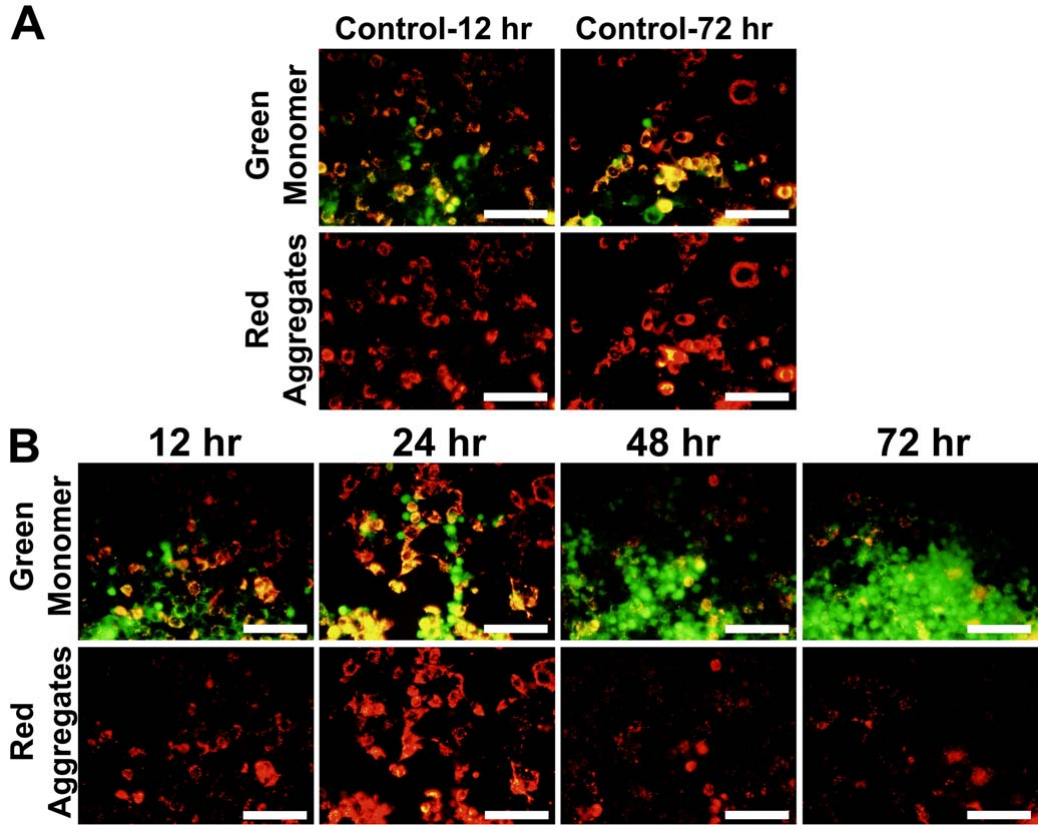
Loss of MMP in Neuro-2a cells correlates with optimum knockdown of Grx1. Cells were transfected with empty vector (control for 12 and 72 hr; (Fig. 2A)) or shRNA to Grx1 (Fig. 2B) and loss of MMP was monitored after 12, 24, 48 and 72 hr of transfection using JC-1 (2 µg/ml) dye. Loss of MMP in response to downregulation of Grx1 was found to be maximum after 72 hr of transfection. Green staining represents JC-1 monomer in cells with loss of MMP whereas red staining represents JC-1 aggregates in cells with intact MMP. Bar represents 120 µm.

We qualitatively examined the mitochondrial membrane potential (MMP) using JC-1 dye in Neuro-2a cells, wherein Grx1 was silenced, after 72 hr after transfection of shRNA to Grx1. JC-1 is a cationic dye which exhibits a potential dependent accumulation in mitochondria which is indicated by a shift in the fluorescence emission. In polarized mitochondria, it accumulates in aggregated form and appears as red punctate staining whereas in cells having depolarized mitochondria, it disseminates into the cytoplasm and appears as green diffused monomeric staining. We observed a time dependent loss of MMP which was maximum at 72 hour post-transfection in Neuro-2a cells (Fig. 2A, B).

### Loss of MMP following Grx1 downregulation

**Figure 3:**
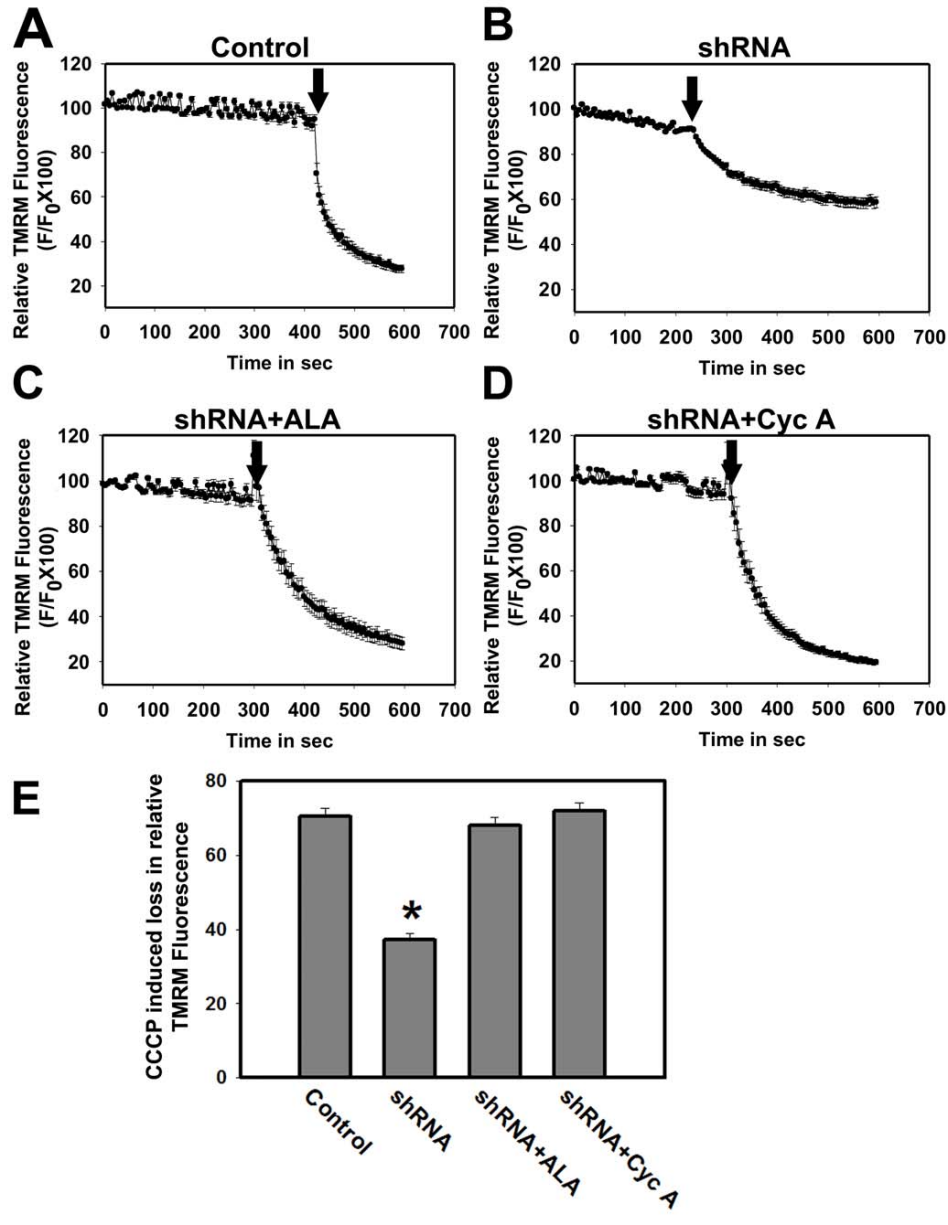
Quantitative determination of loss of MMP in Neuro-2a cells in response to Grx1 knockdown. Grx1 was downregulated in Neuro-2a cells using shRNA to Grx1. Cells were treated with vehicle after transfecting them with empty vector, or with vehicle, α-lipoic acid (100 µM) or cyclosporine A (10 µM), 6 hr after the transfection with shRNA to Grx1. MMP was measured using TMRM as the indicator dye 72 hr after the transfection. Cells were loaded with TMRM and imaged to measure change in TMRM intensity for 300 sec prior to the addition of CCCP. Loss of TMRM intensity was further measured for 300 sec after the addition of CCCP. Cells transfected with empty vector show abrupt decrease in TMRM intensity after CCCP treatment representing sudden loss of MMP (A). Gradual decrease in TMRM intensity in cells transfected with shRNA to Grx1 represents steady loss of MMP even before CCCP treatment which further decays gradually on its addition (B). MMP was maintained in shRNA transfected cells pretreated with α-lipoic acid (C) or cyclosporine A (D). The difference in TMRM fluorescence 2 sec prior and 300 sec after CCCP addition was considered as relative measure of MMP in different groups (E). The data shown are mean±SEM for 25 to 30 cells from 3 independent experiments in each group. Asterisk indicates values significantly different from controls (p<0.05). Loss of MMP due to the Grx1 knockdown is maintained by pretreating the cells with α-lipoic acid and cyclosporine A (E). Arrow represents time point of addition of CCCP.

MMP was quantified by real time imaging of live cells loaded with mitochondrial potential sensitive dye TMRM in Neuro-2a cells. Under normal conditions TMRM sequesters in the polarized mitochondria and shows punctate staining. As the mitochondrial membrane potential is lost, it diffuses out into the cytoplasm and subsequently moves out of the plasma membrane. To quantify the MMP, the fluorescence intensity was measured in several regions of interest (ROI) representing TMRM stained mitochondria in live cells. Under identical dye loading protocol the TMRM fluorescence in cells transfected with shRNA to Grx1 was significantly lower as compared to those transfected with empty vector and did not decrease further with time suggesting that the loss of MMP has already occurred (Fig. 3B,E). To confirm that the decrease in TMRM fluorescence is indeed due to loss of MMP, the protonophore carbonyl cyanide m-chlorophenyl hydrazone (CCCP) was added to depolarize the mitochondrial membrane. CCCP induced an abrupt decrease in TMRM fluorescence reflecting the loss of MMP. The decrease in relative change in fluorescence intensity (ΔF/F) at 300 sec after CCCP addition was considered as the relative measure of MMP. Downregulation of Grx1 resulted in loss of MMP in Neuro-2a cells (Fig. 3B,E) as compared to control cells transfected with empty vector (Fig. 3A,E) and this loss of MMP was abolished by α-lipoic acid (Fig. 3C,E) or cyclosporine A (Fig. 3D,E).

### Perturbation of mitochondrial membrane potential in response to Grx1 downregulation in SH-SY5Y, human neuroblastoma cell line

**Figure 4:**
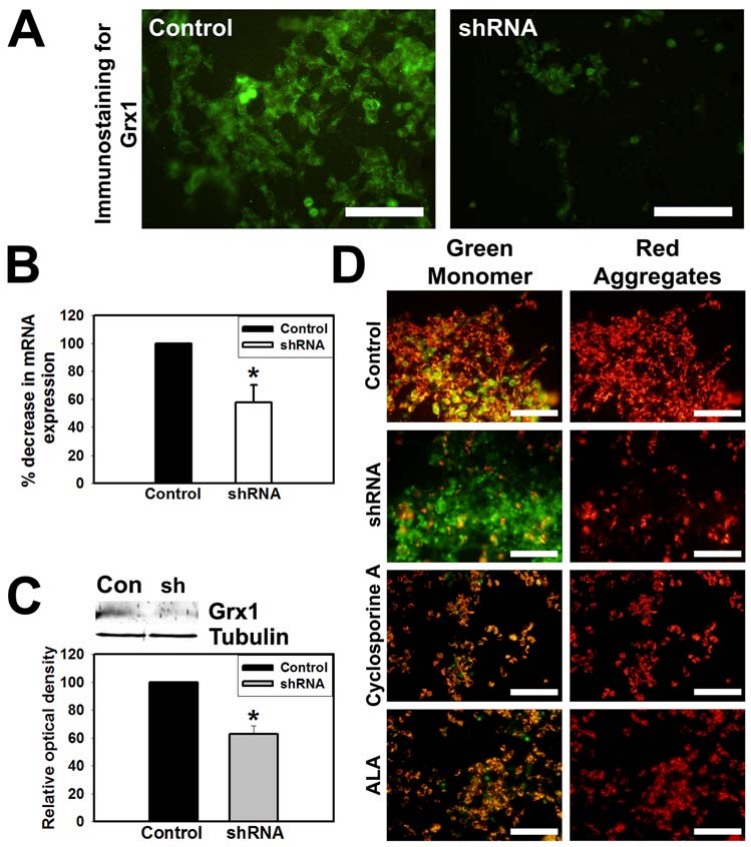
Grx1 silencing causes MMP loss which can be prevented by thiol antioxidants and cyclosporine A. SH-SY5Y cells were transfected with empty vector or shRNA to Grx1 and were used for quantitation of Grx1 at mRNA and protein levels and for the qualitative observation of MMP loss, 72 hr after the transfection. (A) Immunocytochemistry for Grx1 in cells transfected with shRNA to Grx1 show decreased Grx1 levels as compared to the mock control. Bar represents 100 µm. (B) Quantitation of knockdown of Grx1 mRNA levels as assessed by qRT-PCR in SH-SY5Y cells transfected with empty vector or shRNA to Grx1. (C) Immunoblots showing optimal knockdown of Grx1 protein levels in cells transfected with shRNA to Grx1 and densitometric quantitation of immunoblot after normalization with β-tubulin. (D) Loss of MMP was observed in cells transfected with shRNA to Grx1 but not in the cells transfected with empty vector. Pretreatment of cells with cyclosporine A (10 µM) or α-lipoic acid (100 µM) prevented the loss of MMP and only the red aggregates of JC-1 representing healthy cells were observed. Bar represents 120 µm.

We also examined the effects of downregulation of Grx1 in a neuroblastoma cell line of human origin, namely the SH-SY5Y cells, which express estrogen receptors and are sensitive to excitotoxicty. The decrease in levels of Grx1 protein was examined by immunocytochemistry (Fig. 4A), qRT-PCR (Fig. 4B) and immunoblot analysis (Fig. 4C). Downregulation of Grx1 in SH-SY5Y cells resulted in loss of MMP and prior exposure to α-lipoic acid (100 µM), prevented the loss of MMP caused by Grx1 knockdown. Cyclosporine A (10 µM), an inhibitor of mitochondrial permeability transition pore (mPTP) also helped maintain MMP (Fig. 4D) as examined using the JC-1 dye.

### Loss of MMP caused by Grx1 knockdown is rescued by an antioxidant or mPTP blocker

**Figure 5:**
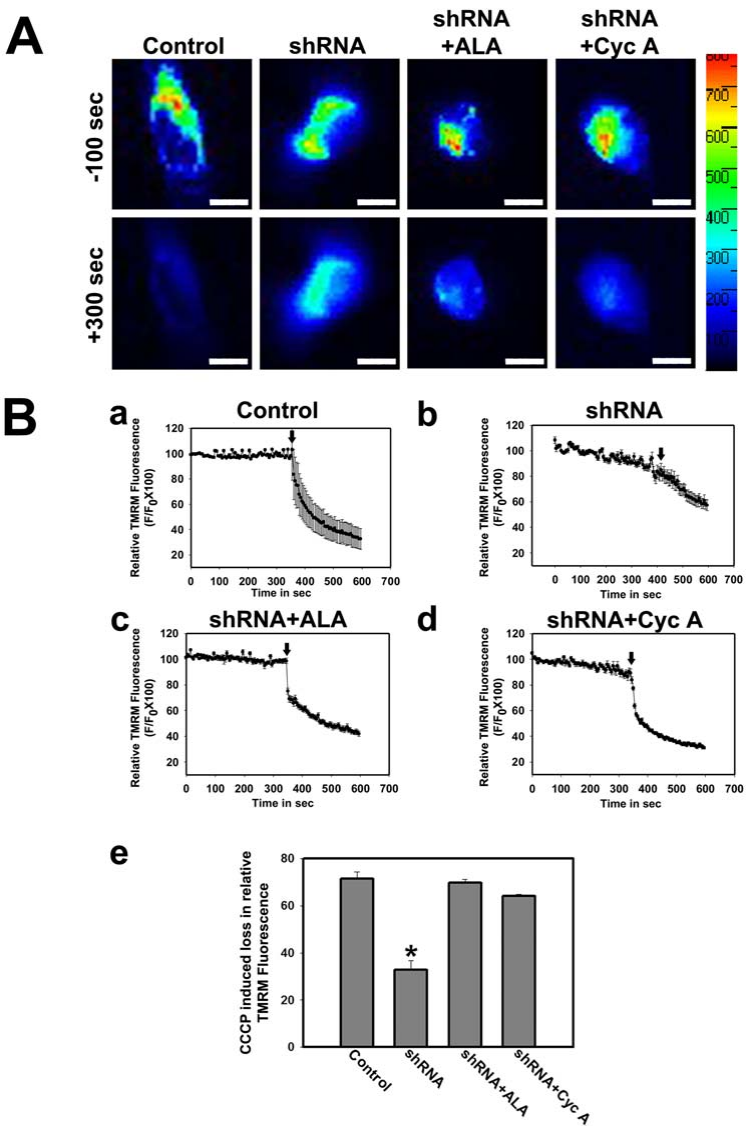
Quantitative determination of loss of MMP in SH-SY5Y cells in response to Grx1 knockdown. Grx1 was downregulated in SH-SY5Y cells using shRNA. Cells were treated with vehicle after transfecting them with empty vector, or with vehicle, α-lipoic acid (100 µM) or cyclosporine A (10 µM), 6 hr post transfection with shRNA to Grx1 and MMP was measured using TMRM as the indicator dye. Cells were loaded with TMRM and imaged to measure change in TMRM intensity for 300 sec prior to the addition of CCCP. Loss of TMRM intensity was further measured for 300 sec after the addition of CCCP. (A) Representative fluorescence images of cells transfected with empty vector or shRNA and treated with either vehicle, α-lipoic acid or cyclosporine A, 100 sec before and 300 sec after addition of CCCP. Bar represents 120 µm. The fluorescence profile in the cell is represented in the pseudocolor bar. (B) Quantification of change in MMP (Δψ_m_). Cells transfected with empty vector show abrupt decrease in TMRM intensity after CCCP treatment representing sudden loss of MMP (a). Gradual decrease in TMRM intensity in cells transfected with shRNA to Grx1 represents steady loss of MMP before CCCP treatment, which further decays on adding CCCP (b). MMP was maintained in shRNA transfected cells pretreated with α-lipoic acid (c) and with cyclosporine A (d). The difference in TMRM fluorescence 2 sec prior and 300 sec after CCCP addition was considered as relative measure of MMP in different groups (e). The data shown are mean±SEM for 25 to 30 cells from 3 independent experiments in each group. Asterisks indicate values significantly different from controls (p<0.05). Loss of MMP caused by Grx1 knockdown is maintained by pretreating the cells with α-lipoic acid (c,e) and cyclosporine A (d,e). Arrow represents time point of addition of CCCP.

Loss of MMP was quantified using TMRM in SH-SY5Y cells. Pseudocolor fluorescence images of TMRM loaded SH-SY5Y cells captured before and after CCCP addition revealed the MMP status in mitochondria of the cells transfected with empty vector or shRNA to Grx1 (Fig. 5A). MMP loss was measured as decrease in TMRM intensity. While there was a sharp drop in the fluorescence in cells transfected with empty vector (Fig. 5B; a,e), similar decrease was not observed in cells transfected with shRNA to Grx1 (Fig. 5B; b,e), confirming that the MMP was already lost in these cells. However, pretreatment of cells with α-lipoic acid (Fig. 5B; c, e) or cyclosporine A (Fig. 5B; d,e) prevented the loss of MMP.

### Cytosolic Grx1 protects against L-BOAA mediated MMP loss and cell death

**Figure 6:**
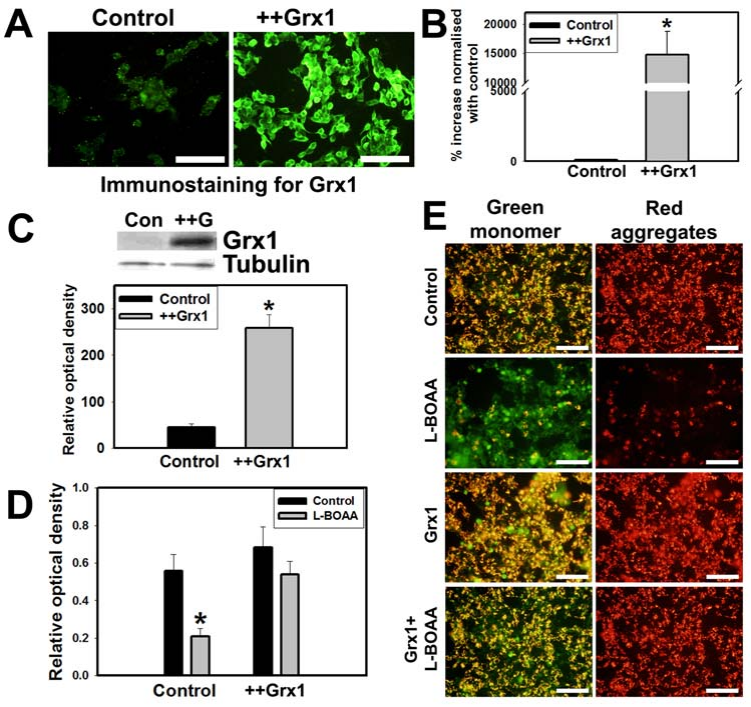
Overexpression of Grx1 prevents loss of MMP and L-BOAA mediated cell toxicity. Grx1 overexpressing SH-SY5Y clonal cell line and control cell lines electroporated with mock empty vector were characterized for the expression of Grx1 by immunostaining (A), quantitation of mRNA by qRT-PCR (B) and immunoblot (C). SH-SY5Y cells stably overexpressing Grx1 and those electroporated with empty vector (control) were exposed to L-BOAA (1 mM) for 24 hr before determining the cell viability. Cells overexpressing Grx1 show more viability after L-BOAA exposure as compared to the control cells (D). Data is represented as mean±SD from 3 independent experiments. Asterisks indicate values significantly different from controls (p<0.05). SH-SY5Y clonal lines overexpressing Grx1 and mock control cells were subjected to L-BOAA (1 mM) treatment for 24 hr before qualitative determination of their MMP status using JC-1 (2 µg/ml). The former (Grx1 overexpressing cell line) maintained MMP following exposure to L-BOAA while control cells showed loss of MMP detected as green JC-1 monomers (E). Bar represents 120 µm.

A stable cell line overexpressing Grx1 was generated by electroporating SH-SY5Y with 20 µg of linearized construct overexpressing Grx1, followed by clonal selection. Overexpression of Grx1 was validated by immunocytochemistry (Fig. 6A), qRT-PCR analysis (Fig. 6B) and immunoblot analysis (Fig. 6C). Overexpression of Grx1 prevented the loss of cell viability seen after L-BOAA (1 mM) treatment indicating that Grx1 offered protection against L-BOAA mediated cell death (Fig. 6D). Exposure of SH-SY5Y cells to L-BOAA (1 mM) resulted in loss of MMP which was not seen in cells overexpressing Grx1 as observed using JC1 dye (Fig. 6E).

### Estrogen upregulates Grx1 and confers protection against L-BOAA toxicity

**Figure 7:**
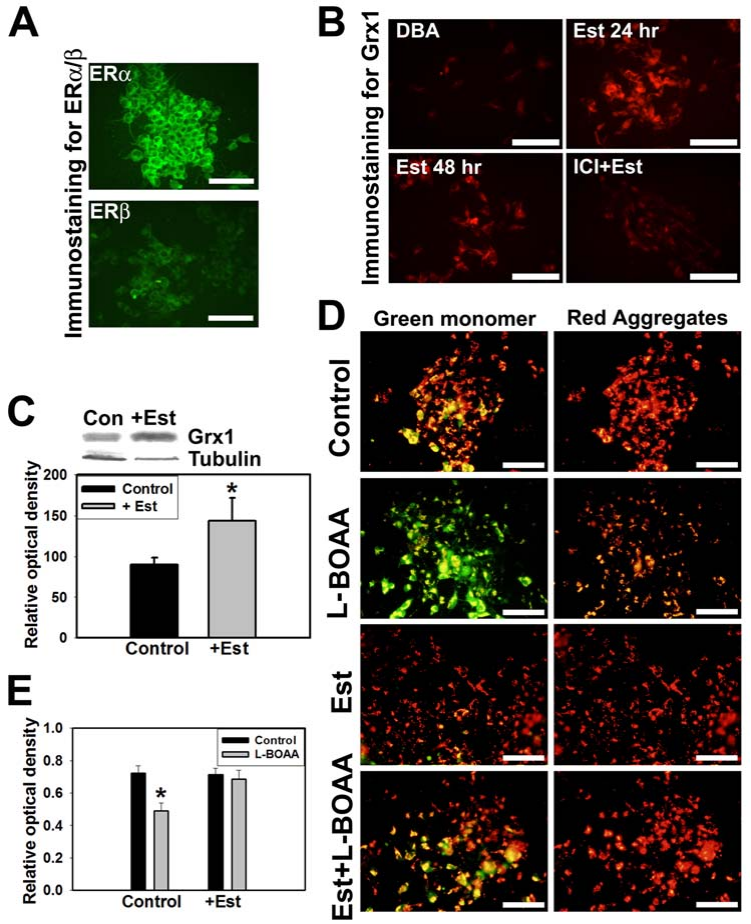
Estrogen upregulates Grx1 and confers protection against L-BOAA toxicity by maintaining MMP. (A) Immunocytochemical staining of estrogen receptors α and β in SH-SY5Y cells. Bar represents 100 µm. (B) SH-SY5Y cells were treated with 17-β estradiol (200 nM) for 24 and 48 hr. One set of cells was pretreated with estrogen receptor antagonist ICI 182780 (1 nM), 1 hr prior to the treatment with 17-β estradiol. Immunostaining for Grx1 shows its upregulation on exposure to 17-β estradiol, which is prevented by ICI 182,780. Bar represents 100 µm. (C) Immunoblot showing upregulation of Grx1 in response to 17-β estradiol (+Est) as compared with control (Con). Densitometric quantitation of immunoblot after normalization with β-tubulin. Data is represented as mean±SD from 3 independent experiments. Asterisks indicate values significantly different from controls (p<0.05). (D) SH-SY5Y cells were exposed to estrogen (200 nM) for 24 hr before treating them with L-BOAA (1 mM; 24 hr) and loaded with JC-1 for monitoring MMP. L-BOAA mediated loss of MMP was abolished in cells pretreated with estrogen as compared to vehicle treated controls. Bar represents 120 µm. (E) Cells were pretreated with 17-β estradiol (200 nM) or vehicle for 24 hrs before exposure to L-BOAA (500 µM; 24 hr). 17-β estradiol protects against L-BOAA mediated cytotoxicity. Data is represented as mean±SD from 3 independent experiments. Asterisks indicate values significantly different from controls (p<0.05).

Grx1 is constitutively expressed in greater amounts in CNS of female mice and ovariectomy downregulates Grx1 rendering them more susceptible to L-BOAA toxicity [21]. Estrogen receptors α and β are expressed in SH-SY5Y cells (Fig. 7A) and treatment with 17-β estradiol upregulated Grx1 as seen by immunostaining (Fig. 7B) and immunoblot (Fig. 7C). To determine if estrogen protects against L-BOAA mediated mitochondrial toxicity we treated the cells with 17-β estradiol (200 nM) 24 hr prior to treatment with L-BOAA (1 mM). MMP was monitored qualitatively using JC-1 dye. Loss of MMP caused by L-BOAA was prevented in the cells pretreated with 17-β estradiol, while cells treated with vehicle were not protected (Fig. 7D). To further confirm the neuroprotection mediated by estrogen we examined the cell viability. Cells were treated with vehicle or 17-β estradiol (200 nM) for 24 hr prior to the treatment with L-BOAA (500 µM) for further period of 24 hr and cell viability assessed. As compared to the vehicle treated cells, those exposed to 17-β estradiol had greater viability indicating that estrogen pretreatment provided protection against L-BOAA mediated toxicity (Fig. 7E).

**Figure 8:**
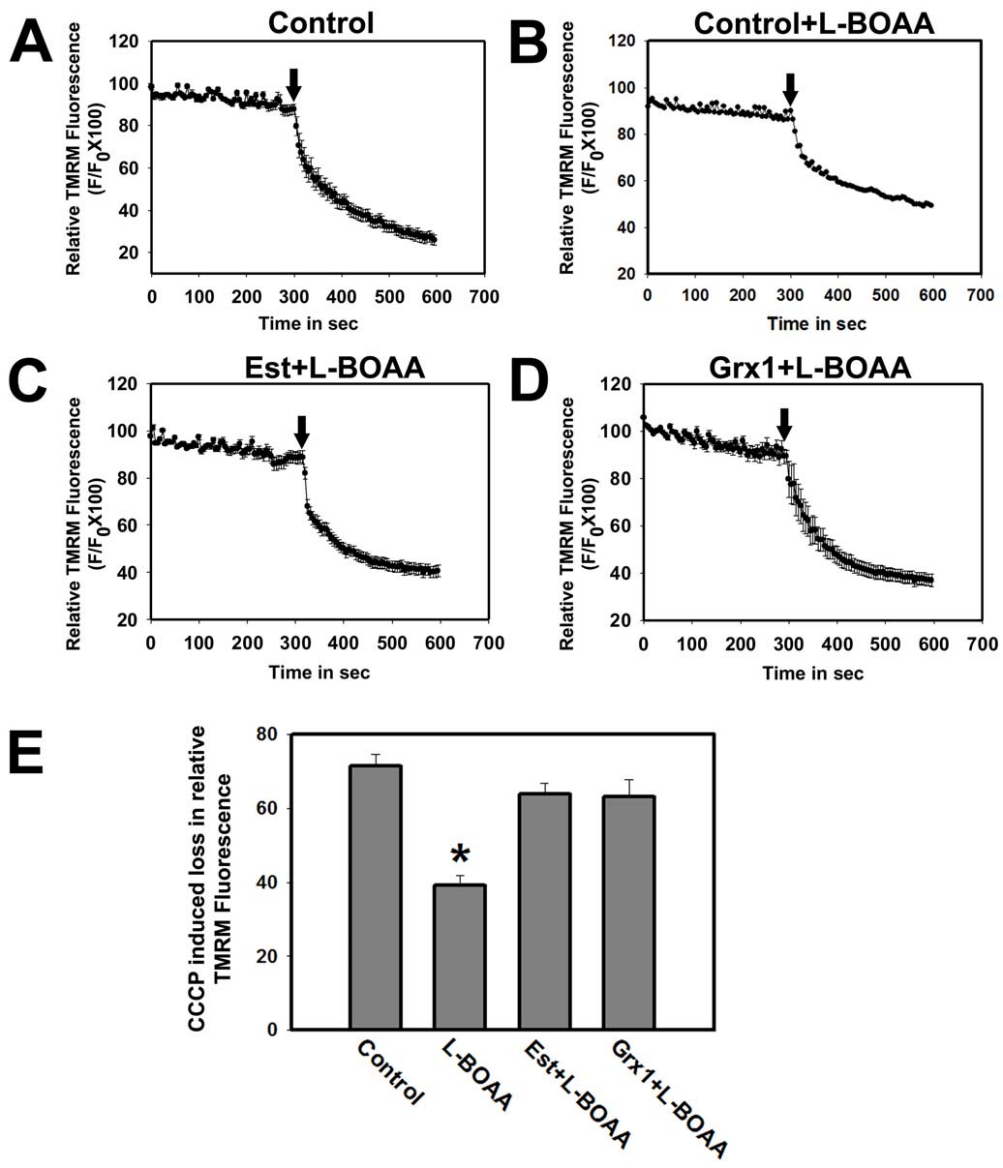
Exposure to estrogen or overexpression of Grx1 abolishes L-BOAA induced MMP loss in SH-SY5Y cells. Cells pretreated with 17-β estradiol (200 nM) and cells stably overexpressing Grx1 were treated with L-BOAA (1 mM; 24 hr). Vehicle treated cells show abrupt decrease in TMRM intensity after CCCP treatment representing sudden loss of MMP (A). Gradual decrease in TMRM intensity in cells treated with L-BOAA represents steady loss of MMP before CCCP treatment, which further decays on adding CCCP (B). MMP is maintained in SH-SY5Y pretreated with 17-β estradiol (C) and in cell lines overexpressing Grx1 (D). L-BOAA mediated loss of MMP is prevented by either pretreating the cells with 17-β estradiol or overexpression of Grx1 (E). Data is represented as mean±SEM from 3 independent experiments. Asterisks indicate values significantly different from controls (p<0.05). Arrow represents time point of addition of CCCP.

Quantitation of loss of MMP using TMRM revealed that cells pretreated with 17-β estradiol maintained MMP (Fig. 8C) like controls (Fig. 8A), as compared to the vehicle treated cells following exposure to L-BOAA (Fig. 8B). The neuroprotection offered by 17-β estradiol was similar to that seen in cells overexpressing Grx1 (Fig. 8D). Therefore, both overexpression of Grx1 or pretreatment of cells with estrogen, which in turn upregulates Grx1, provide protection against L-BOAA mediated toxicity in SH-SY5Y cells (Fig. 8E).

### Oxidation of thiol groups of voltage dependent anion channel (VDAC) by Grx1 knockdown

**Figure 9:**
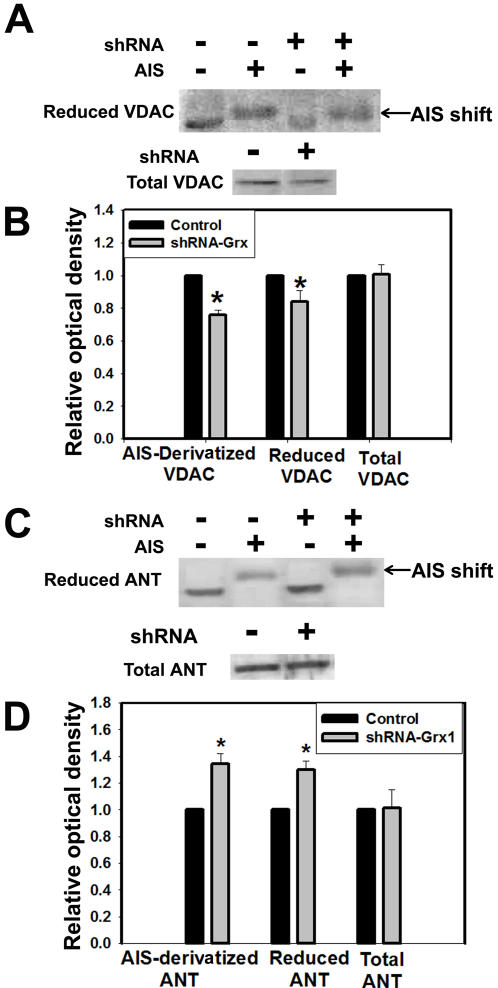
Downregulation of Grx1 causes oxidative modification of thiol groups of voltage dependent anion channel (VDAC). Neuro-2a cells were transfected with shRNA to Grx1 or empty vector and the cell lysates were incubated with AIS which alkylates the free thiol groups thus causing a shift in the migration on non-reducing SDS-PAGE. (A) Immunoblot depicting reduced and AIS derivatized VDAC. Total cell lysate of Neuro-2a cells transfected with empty vector (shRNA ‘−’) or shRNA to Grx1 (shRNA ‘+’) were subjected to non-reducing SDS-PAGE. Reduced VDAC measured as such (AIS ‘−’ ) and as AIS derivatized VDAC (AIS ‘+’), is shown. Total VDAC from control (shRNA ‘−’) or shRNA to Grx1 (shRNA ‘+’) transfected cells was measured using reducing SDS-PAGE followed by immunoblotting. (B) Densitometric measurements of the immunoblots depicted in (A). Reduced and AIS derivatized VDAC were normalized with total VDAC. Values are mean±SD (n = 6) individual experiments. Asterisks indicate values significantly different from controls (p<0.01). (C) Immunoblot depicting reduced and AIS derivatized ANT. Total cell lysate of Neuro-2a cells transfected with empty vector (shRNA ‘−’) or shRNA to Grx1 (shRNA ‘+’) were subjected to non-reducing SDS-PAGE. Reduced ANT measured as such (AIS ‘−’) and as AIS derivatized ANT (AIS ‘+’), are depicted. Total ANT from control (shRNA ‘−’) or shRNA to Grx1 (shRNA ‘+’) transfected cells was measured using reducing SDS-PAGE followed by immunoblotting. (D) Densitometric measurements of the immunoblots shown in (C). Reduced and AIS derivatized ANT were normalized with total ANT. Values are mean±SD (n = 6) individual experiments. Asterisks indicate values significantly different from controls (p<0.01).

Grx1, a cytosolic enzyme appears critical for maintenance of mitochondrial integrity; however the underlying mechanism is unclear. Grx1 is an oxido-reductase and is involved in maintaining the redox status of several redox sensitive proteins. We therefore hypothesized that Grx1 may participate in a crosstalk across the mitochondrial membrane through the modification of mitochondrial membrane proteins. VDAC and ANT are components of mPTP that are present in the outer and inner mitochondrial membranes, respectively. They are redox sensitive proteins and can be potentially modulated by Grx1 by altering the redox status of their thiol groups. Neuro-2a cells were transfected with empty vector or shRNA to Grx1 and cells were collected after 72 hr and incubated (derivatized) with AIS, an alkylating agent that binds to free thiol groups in proteins. AIS derivatized samples were separated by electrophoresis under non-reducing conditions and subjected to immunoblotting using antibody to VDAC and ANT. We observed significantly lower amounts of AIS derivatized (reduced) VDAC in cells wherein Grx1 was knocked down as compared to cells transfected with vector alone (Fig. 9A), while total VDAC levels (reduced plus oxidized) were unchanged indicating that VDAC protein was oxidatively modified by Grx1 knockdown. Moreover, ANT, an inner membrane mitochondrial protein did not show oxidative modification upon downregulation of Grx1 and the reduced state of ANT was increased significantly (Fig. 9B), indicating that Grx1 downregulation oxidatively modifies the redox state of outer membrane but not inner membrane proteins in the mitochondria.

## Discussion

During oxidative stress GSH is oxidized to GSSG (eqn. 1), which is often effluxed out of the cell thereby preventing the oxidative modification of protein thiols (PrSH) to protein glutathione mixed disulfide (PrSSG; eqn. 2). Protein thiols can also be directly oxidized by ROS to thiyl radicals or in a sequential manner to sulfenic, sulfinic and sulfonic acid (eqn. 3). Sulfenic acids can react with GSH to form PrSSG thus preventing their irreversible oxidation to sulfonic acids (eqn. 4). PrSSG may be further modified to generate intramolecular and intermolecular protein mixed disulfides (PrSSPr; eqn. 5). PrSSGs are reduced back to protein thiols very effectively by Grx1 (in the cytosol) and Grx2 (in the mitochondria) utilizing GSH and reducing equivalents of NADPH (eqn. 6). The GSSG generated by this reaction is reduced back to GSH by glutathione reductase using reducing equivalents from NADPH (eqn. 7). Downregulation of Grx1 would therefore potentially lead to increased levels of protein glutathione mixed disulfides as also mixed disulfides of proteins (PrSSPr). In addition, generalized increase in ROS in the cell caused by Grx1 knockdown (Fig. 1) could lead to other non-specific oxidative events.
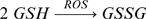
(1)

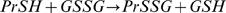
(2)

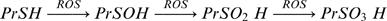
(3)

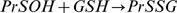
(4)

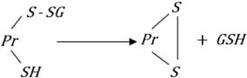
(5)

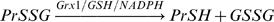
(6)

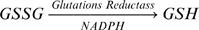
(7)


**Figure 10:**
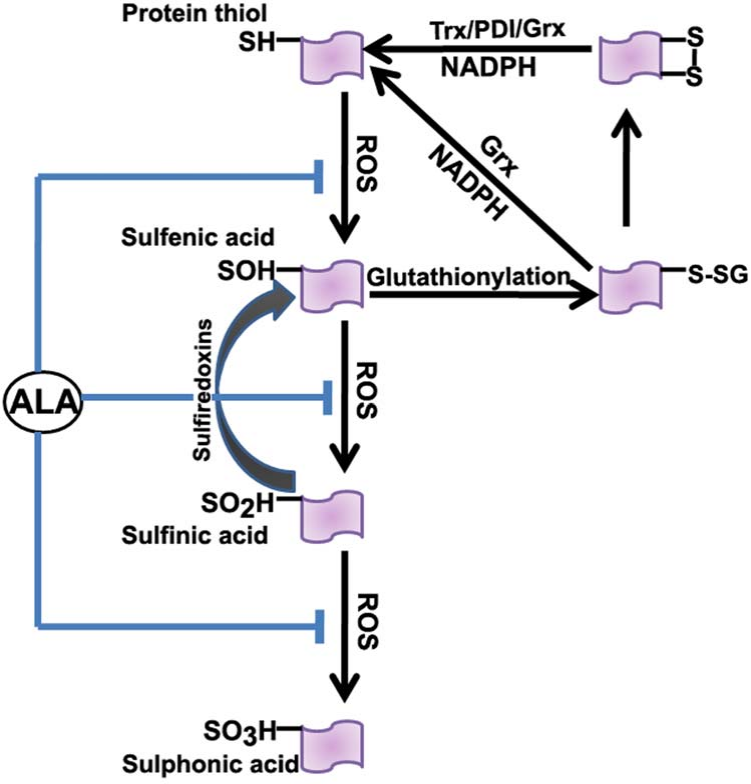
Oxidative modification of thiol groups in proteins: Protein thiols may be oxidized sequentially to sulfenic, sulfinic and sulfonic acid. Sulfenic acids can react with GSH to form PrSSG thus preventing their irreversible oxidation to sulfonic acids. PrSSG may be further modified to protein mixed disulfides. PrSSG are reduced back to protein thiols very effectively by Grx1 utilizing GSH and reducing equivalents of NADPH. The thiol antioxidant, α-lipoic acid, can potentially prevent the oxidative modification of protein thiols.

In the present study we demonstrate that the cytosolic protein disulfide oxido-reductase Grx1, plays an important role in the maintenance of mitochondrial function and downregulation using shRNA to Grx1 dramatically alters the mitochondrial membrane potential which can be restored by α-lipoic acid and cyclosporine A. α-Lipoic acid, an endogenous thiol antioxidant was very effective in preventing MMP loss mediated by Grx1 downregulation. The ability of α-lipoic acid to afford protection against the MMP loss could be due to the fact that the oxidative modification of protein thiols is restricted to the formation of sulfenic acid and mixed disulfides which can be reversed enzymatically by sulfiredoxins and glutaredoxin, respectively [31]–[34]. If oxidative modification of protein thiols proceeded from the formation of sulfinic acid(s) [35] to the sulfonic acid derivatives, it would be irreversible. (Fig. 10). This effect of downregulation of Grx1 was noted in two neuronal cell lines, SH-SY5Y and Neuro-2a of human and mouse origin respectively.

MMP is regulated by critical proteins in the mitochondria, such as VDAC, ANT and cyclophilin. The redox status of these proteins are known to affect the MMP, for example, the oxidative state of the vicinal thiol groups in VDAC are known to critically affect MMP and the opening of mitochondrial permeability transition pore (mPTP) [36]. Similarly the redox status of ANT is also known to be crucial for the maintenance of MMP [37]–[39]. We, therefore examined the redox status of both VDAC and ANT following downregulation of Grx1 and found that while there was a dramatic loss of reduced VDAC, ANT did not show a similar loss. Interestingly there was an increase in the amount of reduced ANT measured both as the derivatized and the reduced protein. This could be due to the fact that ANT is unaffected by downregulation of Grx1 or the mitochondrial glutaredoxin Grx2, efficiently reduces oxidized ANT. Although the expression of Grx2, the mitochondrial form of glutaredoxin located predominantly in the matrix was unchanged when Grx1 was downregulated (Fig. 1B), its potential role in maintaining the reduced state of key mitochondrial proteins cannot be ruled out.

A recent report has demonstrated the presence of Grx1 in the intermembrane space in the mitochondria [40]. The knockdown of Grx1 should potentially downregulate the expression of Grx1 both in the cytosol and the intermembrane space of the mitochondria, although it is presumable that the turnover rate of Grx1 could be different in the cytosol and the intermembrane space of the mitochondria and therefore knockdown of Grx1 may not be similar in the two subcellular compartments. This may partly explain the lack of oxidative modification of the cysteine residue(s) in ANT. The role of Grx1 in redox regulation in the intermembrane space is yet to be clearly understood and additional studies are needed in this direction.

Our results point to the fact that the oxidative modification of VDAC by downregulation of Grx1 could be a major player in mediating the loss of MMP. Several forms of VDAC are known to exist, some of which may not have any effect on MMP [41]. However, others have provided evidence for the importance of VDAC in maintenance of MMP [42]–[45]. We demonstrate for the first time that perturbation of the redox milieu in the cytosol through knockdown of Grx1 can modify VDAC which could potentially result in the observed loss of MMP. Our results also point that even when the reduced form of ANT is not decreased, perturbation in VDAC could lead to MMP loss. Since the reduced form of the inner membrane protein ANT did not decrease by knockdown of Grx1, we did not monitor the redox status of cyclophilin D, which resides in the mitochondrial matrix.

L-BOAA is an excitatory amino acid that causes mitochondrial dysfunction [12], [21]. Exposure of SH-SY5Y cells to L-BOAA resulted in loss of MMP. However, in cells overexpressing Grx1, L-BOAA was unable to adversely affect MMP or cell viability. Grx1 expression is regulated by estrogen in the central nervous system (CNS) and overiectomy downregulates Grx1 levels in brain regions [46]. We were able to detect this phenomenon in SH-SY5Y cells which express both α and β estrogen receptors. Exposure of cells to estrogen upregulated Grx1 and prevented L-BOAA mediated loss of MMP clearly delineating the important role of Grx1 in maintaining mitochondrial function and thereby the cell viability.

**Figure 11:**
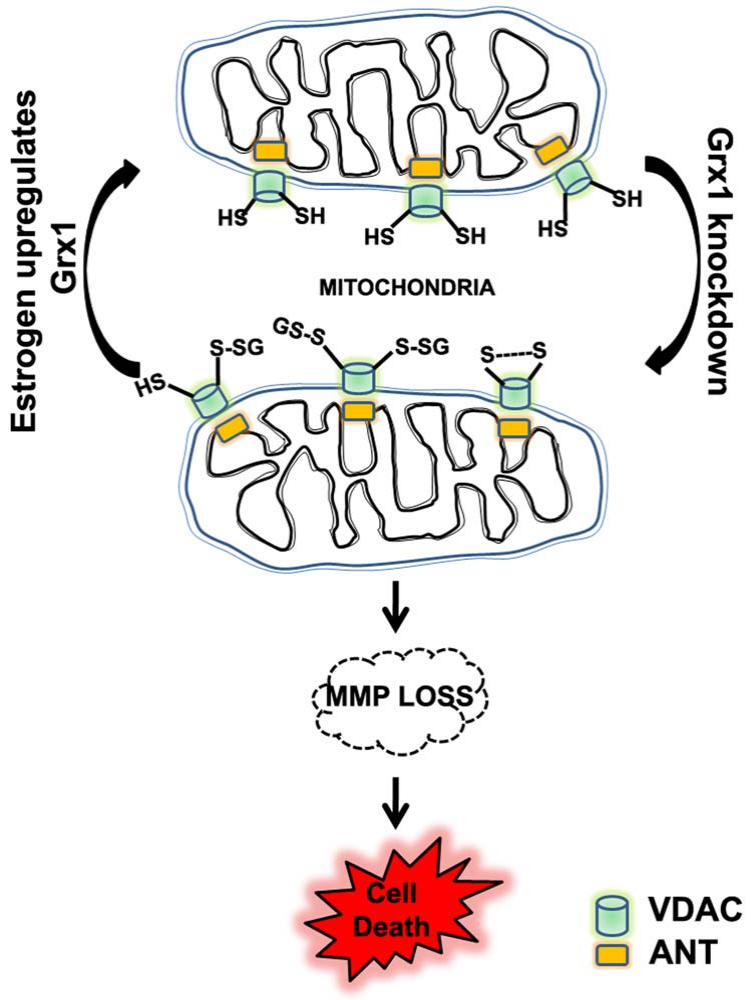
Cytosolic Grx1 downregulation results in loss of mitochondrial membrane potential: Downregulation of cytosolic Grx1 leads to modification of critical thiol groups of the outer mitochondrial membrane protein VDAC, resulting in loss of membrane potential which could eventually lead to cell death. Grx1 expression is regulated by estrogen and its upregulation by estrogen prevents MMP loss by maintaining redox status of critical thiol groups in the mitochondria.

In conclusion, we demonstrate that the downregulation of the cytosolic Grx1 can lead to mitochondrial dysfunction detected as loss of MMP which occurs through redox modification of VDAC but not ANT in the cell lines examined (Fig. 11). Maintenance of protein thiol homeostasis through Grx1 may play an important role in disease pathogenesis such as Parkinson's disease and motor neuron disease, wherein mitochondrial dysfunction is a key player.

## Materials and Methods

L-BOAA was obtained from Research Biochemicals (Natick, MA). ICI 182,780 and JC-1 were obtained from Tocris Cookson (Avonmouth, UK) and Molecular Probes respectively (Eugene, Oregon). Tetramethyl rhodamine methyl ester (TMRM) and MitoTracker were purchased from Molecular Probes (Eugene, OR, USA); carbonyl cyanide *m*-chlorophenylhydrazone (CCCP) from Aldrich (Milwaukee, WI, USA). Antibody to Grx1 and Grx2 were obtained from Lab Frontiers Life Science Institute (Seoul, Korea). Antibody to estrogen receptors α and β were obtained from Santa Cruz Biotechnology Inc., CA. Cell culture products and Lipofectamine™ were obtained from Gibco BRL (Invitrogen, Carlsbad, CA). All other chemicals and reagents were of analytical grade and were obtained from Sigma Chemical Company (St. Louis, MO) or Merck (Darmstadt, Germany).

### Cells

SH-SY5Y and Neuro-2a cells (American Type Culture Collection, Manassas, VA, USA) were cultured in MEM and DMEM medium, respectively, supplemented with 10% (v/v) fetal bovine serum, 100 units/ml penicillin G and 100 mg/ml streptomycin. Stable cell lines overexpressing Grx1 and control cell lines transfected with empty vector, were developed by electroporating SH-SY5Y cells with 20 µg of pcDNA-pCMV-Grx1 and pcDNA-3.1 each respectively with a single pulse of 1 ms at 200 V, followed by clonal selection and expansion. Stable cell lines were maintained with complete MEM supplemented with 500 µM Geneticin.

### Treatment of cells

Regulation of Grx1 by estrogen was studied in SH-SY5Y cells following differentiation with dibutyryl cyclic AMP (DBA; 1 mM) for 24 hr. Cells were treated with 17-β-Estradiol (200 nM) for 24 hr further. In some experiments cells were exposed to ICI 182,780 (1 nM), 30 min prior to exposure to estrogen. Differentiated cells pretreated with estradiol or vehicle were treated with L-BOAA (1 mM) for a further period of 24 hr and used for different experiments. In some experiments involving the measurement of MMP, α-lipoic acid (100 µM) or cyclosporine A (10 µM) were added 6 hr after transfection or along with L-BOAA.

### Cell viability assay

MTT (3-(4, 5-dimethylthiazolyl-2)-2, 5-diphenyltetrazolium bromide; 5 mg/ml in PBS) was added at a final concentration of 125 µg/ml after treatment or transfection. Cells were incubated with MTT for 3 hr at 37°C, solubilized in dimethyl formamide (50%; v/v) and SDS (20%; w/v), prior to measurement of absorbance at 570 nm.

### Downregulation of Grx1

The following oligonucleotide sequences were annealed and cloned into mU6pro vector kindly provided by Prof. D. Turner, (Univ. of Michigan, Ann Arbor): 5′-TTTGCGGATGCAGTGATCTAATAAGTTCTCTATTAGATCACTGCA-TCCGCTTTT–3′, 3′GCCTACGTCACTAGATTATTCAAGAGATAATCTAGTCACGTAGGCGAAAAAGATC - 5′. The construct was then transfected using Lipofectamine™2000 (Invitrogen) into Neuro-2a or SH-SY5Y as required according to the instructions provided by the manufacturer. Since there is 87% homology between mouse and human Grx1, the same shRNA sequence could be used to downregulate Grx1 expression in cells of both murine and human origin (Fig. S1).

### Immunocytochemistry

SH-SY5Y and Neuro-2a cells were seeded in chamber slides and fixed with 4% paraformaldehyde (w/v) for each experiment.

Localization of Grx1 was done by loading Neuro-2a cells with Mito Tracker Deep Red 633 (500 nM) as per manufacturer's instruction and then fixed with 3.7% formaldehyde (v/v). Cells were then immunostained using antiserum to Grx1 (1∶500) and Grx2 (1∶500) overnight at 4°C and incubated with secondary antibody conjugated to Alexa Flour 488 for 1 hr and visualized under fluorescence microscope. Cells were also immunolabeled for α and β estrogen receptors.

### Intracellular ROS measurement

The oxidant sensitive dye H_2_DCFDA (2′,7′-dichlorodihydrofluorescein diacetate) was used to measure intracellular ROS. Cells were seeded at 60% confluency 24 hr prior to transfection with shRNA to Grx1 or empty vector. After 72 hr of transfection, Neuro-2a cells were loaded with 10 µM H_2_DCFDA dissolved in dimethyl sulfoxide and incubated for 15 min at 37°C. Cells were then washed with PBS and imaged using appropriate filter in an inverted fluorescence microscope.

### Quantitative real-time PCR for assessing Grx1 expression

Total RNA was isolated from cells transfected with shRNA to Grx1, empty vector and stable cell lines overexpressing Grx1 and used for cDNA synthesis using random hexamers. Quantitative real time PCR was done for mouse (Mm) and human (Hs) Grx1 using specific primers stated as - Mm-Grx1-forward 5′-TCCTCAGTCAACTGCCTTTCA-3′, Mm-Grx1-reverse 5′-CTCCGGTGAGCTGTTGTAAA-3′ and Hs-Grx1-forward 5′-TCGATATCACAGCCACCAAAC-3′, Hs-Grx1-reverse 5′-CACTGCATCCGCCTATACAA-3′ respectively. 18S rRNA was used as internal control for normalization.

### Qualitative assessment of MMP using JC-1

JC-1 (5,5′,6,6′-tetrachloro-1,1′,3,3′-tetraethylbenzimidazolcarbocyanine iodide) is sensitive to mitochondrial membrane potential and the shift in membrane potential is observed as a decrease in ratio of red to green intensity with the loss of mitochondrial membrane potential. After the desired treatment, cells were loaded with JC-1 (2 µg/ml) for 30 min at 37°C in the culture medium. The fluorescence intensity of both monomer (green) and aggregated (J-aggregates, red) molecules were observed immediately using appropriate filters.

### Quantitation of mitochondrial membrane potential by live cell imaging

Mitochondrial membrane potential was assessed using lipophilic cationic fluorescent probe, tetramethyl rhodamine methyl ester (TMRM) that localizes into the mitochondria as a function of membrane potential. Cells grown on coverslips were loaded with 500 nM TMRM for 30 min at 37°C in Hank's balanced salt solution. Real time imaging was performed with a fluorescence imaging system with a monochromatic light source (TILL Photonics, Germany) as described earlier [47]. Dye loaded cells were maintained in a perfusion chamber (bath volume = 0.5 ml) mounted on the microscope stage. Fluorescence images were recorded every 5 sec using 520 nm excitation and 2×2 on-chip binning. Quantification of mitochondrial membrane potential was performed as described earlier [47]. Regions of interest (ROI) were selected within several cells in each experiment for measuring change in mitochondrial membrane potential (MMP). To quantify the MMP, protonophore CCCP was added to depolarize the mitochondrial membrane and the difference in fluorescence intensity 10 sec before and 300 sec after CCCP addition was considered as the relative measure of MMP. Data is represented as percent relative change in TMRM fluorescence (F/F_0_)X100, where F_0_ and F are the fluorescence intensities at the beginning of recording and at given time points, respectively.

### Redox state of voltage dependent anion channel (VDAC) and adenine nucleotide translocase (ANT) in Grx1 knocked down cells

Cells were washed with ice cold PBS, harvested and treated with lysis buffer (1× PBS, 0.5% Igepal v/v). The lysates from control cells and those transfected with shRNA-Grx1 were incubated (derivatized) with 4-acetamido-4′-((iodoacetyl)amino)stilbene-2,2′-disulfonic acid disodium salt (AIS; 30 mM; Invitrogen, CA) in Tris buffer (20 mM, pH 7.5), at 25°C for 12 hr and subjected to non-reducing SDS-PAGE, such that the free thiols in the reduced proteins were alkylated with AIS and thus could be visualized as a shift in the blot. The samples that were not derivatized with AIS were subjected to both non-reducing and reducing SDS-PAGE, followed by immunoblotting using antibody to VDAC or ANT.

### Immunoblot analysis

Cells were washed with PBS and treated with lysis buffer [50 mM Tris, (pH 7.4), containing NaCl (150 mM), Triton X-100 (1%; v/v), deoxycholic acid sodium salt (1%; w/v), SDS (0.1%) and EDTA (1 mM)]. Protein concentration was estimated and lysates were subjected to SDS-PAGE, transferred to PVDF membrane, and incubated with the antibody to Grx1, followed by incubation with anti-rabbit IgG labeled with alkaline phosphatase or horse-radish-peroxidase. Immunostained bands were detected using nitroblue tetrazolium and 5-bromo-4-chloro-3-indolyl phosphate as chromogens or using chemiluminescence kit (ECL, Amersham Pharmacia Biotech, France). All blots were normalized with β-tubulin.

Statistical analysis was performed using student ‘t’ test, or ANOVA followed by Student-Newman Keul's or Dunnett's test as appropriate.

## Supporting Information

Figure S1 Sequence alignment of mouse (Mus musculus) and human, (Homo sapiens) cDNA to Grx1 using ClustalW: Sequence alignment shows 87% homology. The highlighted sequence was used for generating shRNA, which shows 95% similarity between mouse and human Grx1.
(7.06 MB DOC)
