# Supplementary figures and images for: Knockdown of Cytosolic Glutaredoxin 1 Leads to Loss of Mitochondrial Membrane Potential: Implication in Neurodegenerative Diseases
Source: PLoS One. 2008 Jun 18;3(6):e2459. doi: 10.1371/journal.pone.0002459 (PMC2426930; doi:10.1371/journal.pone.0002459)

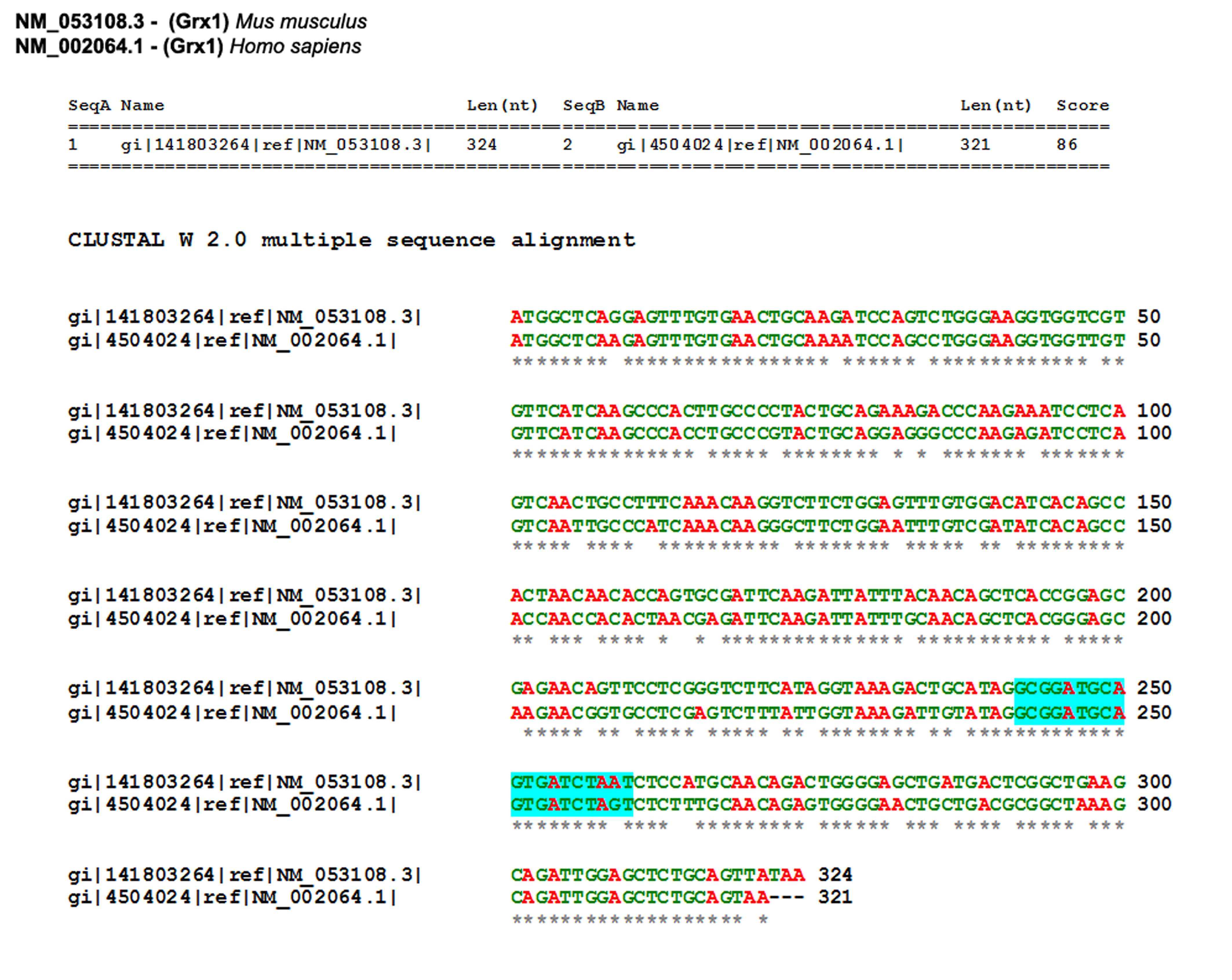

Supplement: Figure S1 — Sequence alignment of mouse (Mus musculus) and human, (Homo sapiens) cDNA to Grx1 using ClustalW: Sequence alignment shows 87% homology. The highlighted sequence was used for generating shRNA, which shows 95% similarity between mouse and human Grx1. (7.06 MB DOC) [file pone.0002459.s001.tif]
